# Computational Forecasting Methodology for Acute Respiratory Infectious Disease Dynamics

**DOI:** 10.3390/ijerph17124540

**Published:** 2020-06-24

**Authors:** Daniel Alejandro Gónzalez-Bandala, Juan Carlos Cuevas-Tello, Daniel E. Noyola, Andreu Comas-García, Christian A García-Sepúlveda

**Affiliations:** 1 Engineering Faculty, UASLP, San Luis Potosí 78290, Mexico; danielgoba84@gmail.com; 2 Commerce and Administration Faculty, UAT, Tamaulipas 87000, Mexico; 3 Microbiology Department, Medicine Faculty, UASLP, San Luis Potosí 78290, Mexico; dnoyola@uaslp.mx (D.E.N.); andreu.comas@uaslp.mx (A.C.-G.); 4 Viral and Human Genomics Laboratory, Medicine Faculty, UASLP, San Luis Potosí 78290, Mexico; christian.garcia@uaslp.mx

**Keywords:** bioinformatics, epidemics, data science, artificial intelligence, pattern recognition, forecasting, outbreaks

## Abstract

The study of infectious disease behavior has been a scientific concern for many years as early identification of outbreaks provides great advantages including timely implementation of public health measures to limit the spread of an epidemic. We propose a methodology that merges the predictions of (i) a computational model with machine learning, (ii) a projection model, and (iii) a proposed smoothed endemic channel calculation. The predictions are made on weekly acute respiratory infection (ARI) data obtained from epidemiological reports in Mexico, along with the usage of key terms in the Google search engine. The results obtained with this methodology were compared with state-of-the-art techniques resulting in reduced root mean squared percentage error (RMPSE) and maximum absolute percent error (MAPE) metrics, achieving a MAPE of 21.7%. This methodology could be extended to detect and raise alerts on possible outbreaks on ARI as well as for other seasonal infectious diseases.

## 1. Introduction

Acute respiratory infections (ARI) are one of the main causes of morbidity and mortality in the world, particularly in children under 5 years old and adults over 65 years old. It has been estimated that 156 million acute lower respiratory infections occur worldwide every year and almost 2.4 million deaths are estimated to have occurred associated with these infections in 2016 [1,2,3,4,5]. Therefore, the development of an effective monitoring and response system for infectious diseases is still a challenge [6,7].

The most frequent pathogens that cause ARI are respiratory syncytial virus (RSV), human metapneumovirus, rhinovirus/enterovirus, influenza viruses, parainfluenza 1–4, adenovirus, coronavirus, *Streptococcus pneumoniae*, and *Mycoplasma pneumoniae* [8,9]. ARI exhibit a seasonal pattern where RSV and influenza viruses are the major contributing pathogens. Changes in circulating viral strains of these viruses may result in yearly winter ARI epidemics. In addition, the introduction of novel influenza strains or other viruses into the human population can lead to the emergence of pandemics.

Despite the health and economic burden of RSV, there is currently no vaccine or effective antiviral tretament against this virus. In contrast, there are several antivirals and vaccines available for influenza. While mortality associated with influenza has been reduced since the introduction of influenza vaccine, this virus remains an important cause of ARI [10].

Health surveillance around the world has become a subject of primary concern, due to the continuous emergence of infectious disease outbreaks, including those associated with ARI such as pandemic influenza, severe acute respiratory syndrome (SARS), and more recently, SARS-CoV-2 [11]. A detailed understanding of transmissible disease dynamics and the accurate forecast of disease outbreaks translate in reductions in the loss of human lives and money savings derived from avoiding desperate measures during health contingencies [12,13,14].

Several systems which collect epidemiological information from informal sources like non-government reports, news reports and field agents have been proposed as potential Early Warnings Systems (EWS). ProMED [15], GOARN [16], GPHIN [17], Argus [18], BioCaster [19], EpiSPIDER [20,21], PREDICT [22] are some examples. ProMED proved the usefulness of EWS with the early report of the SARS epidemic in 2003 in mainland China. In February 10, 2003, a ProMED report became the earliest public alert of a disease which would later be known as severe acute respiratory syndrome (SARS) and which would ultimately affect in excess of 8000 individuals worldwide and kill more than 900 [23].

The use of Inernet search engines by the general public and physicians creates trends of terms, which match the temporal occurrence of diseases and allow for potential detection of outbreaks at early stages and before traditional surveillance methods identify them [24,25]. In the past years, the use of Internet search engines and social media platforms for surveillance and forecast of diseases has been widely studied. Google [26,27,28,29,30,31,32,33,34,35,36,37,38], Yahoo [39], Baidu [26,36,40], and Twitter [31,32] are some of the services that have been used to this effect. Infectious diseases such as dengue [25,36,41,42], influenza-like-illnesses (ILI) [24,27,31,32,33,35,39,40,43,44,45,46], diarrhea [47], varicella [29,47], Lyme disease [28], and Zika [37] are among those that have been studied more frequently with this approach due to their seasonal and epidemic behaviors.

The related work to this research includes Santillana et al. [32], in 2015. They used six different data sources to predict 2013 and 2014: CDC-reported ILI, near real-time hospital visit records from athenahealth, Google trends, influenza related Twitter microblogging, FluNearYou, and Google Flu Trends. One data source is unavailable since 2015, Google Flu Trends. The predictions are made by three different machine learning algorithms to perform multivariate regression, including stacked linear regression, support vector machines (SVM) and AdaBoost with decision tree regression. They report that they are able to predict one, two, and three weeks in the future. This research only reports different data sources and different regression methods, and the performance is evaluated with the Pearson correlation, root mean squared error (RMSE), maximum absolute percentage error (MAPE), and hit rate [32]. Additionally, Volkova et al. [31], in 2017, employed only two data sources: Defense Medical Information System (in USA) and Twitter (period 2011 to 2014). They use as baseline models SVM and AdaBoost and they proposed a long short-term memory (LSTM), which is a recurrent neural network. They trained the LSTM models on two seasons (2012–2013) and tested on the 2014 season. They also employed the same metrics used by Santillana et al. [32], except the hit rate. Their models are capable of predicting weekly ILI dynamics and forecasting up to several weeks in advance [31].

Therefore, previous research only report what data has been used and the best regression models for a specific period of time. Little has been said about data retrieval, data preprocessing, and feature extraction (called data acquisition process). They also do not use endemic channel information.

The main contribution of this research is a methodology composed of the data acquisition and the computational model. Other contributions of this research include: (i) a method to automatically select the search terms associated to the data source available; (ii) a smoothed endemic channel calculation; (iii) a predictive calculation made by merging the forecasting of an artificial neural network, the projection of a sum of sines model, and the proposed smoothed endemic channels.

Overall, this paper presents a methodology capable of making accurate predictions of ARI activity with data obtained from epidemiological reports along with search terms usage derived from the Google search engine. The combined use of epidemiological, machine learning, and forecasting techniques allowed us to develop a computational model that is capable of accurately predicting ARI trends. Adaptations of this model might prove useful for timely detection of outbreaks at early stages and before they become a major health burden. This research is part of a bigger project called Mexican Infectious Disease Analysis and Surveillance mapping application (MIDASmap; http://midasmap.uaslp.mx/) which is under development and will be available online.

## 2. Materials and Methods

**Figure 1:**
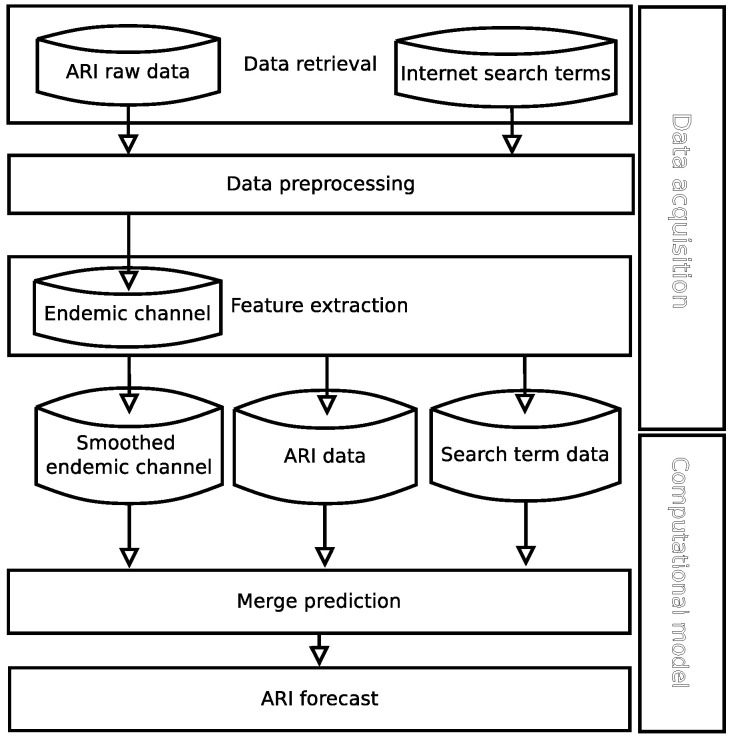
The Proposed Methodology is composed by two stages: the data acquisition and the computational model.

**Figure 2:**
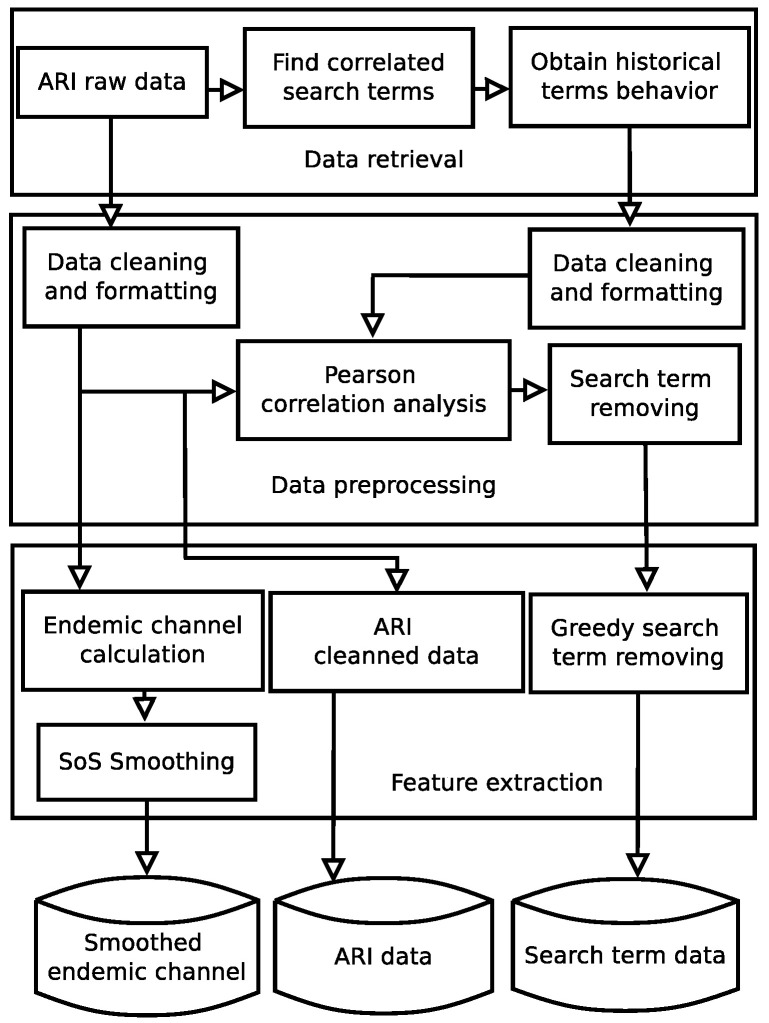
The Data acquisition stage is composed by three modules: Data retrieval, data preprocessing and feature extraction.

**Figure 3:**
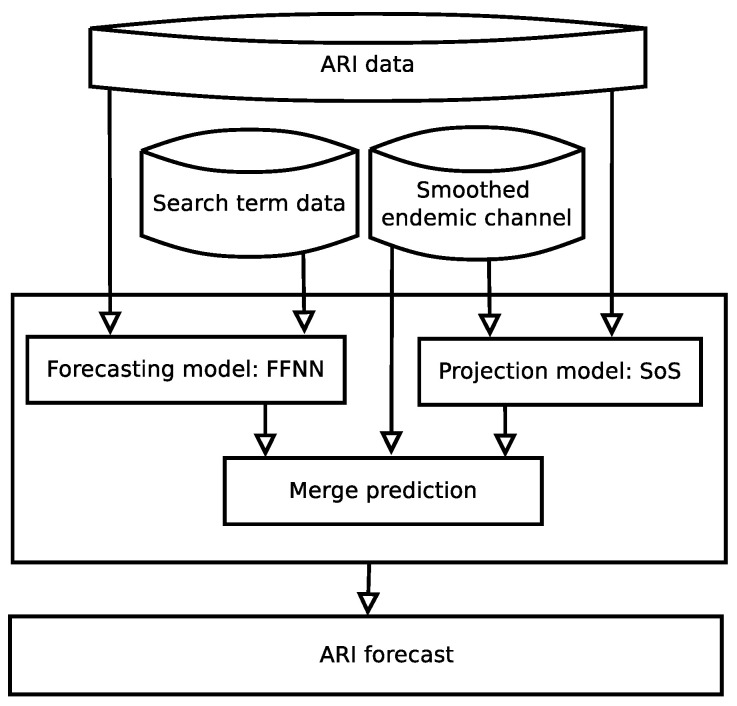
The Computational model, which receives the ARI data, the search terms data and the smoothed endemic channel data as inputs, and is composed by a Forecasting model working in parallel with a Projection model to feed the third module of this stage, the Merge prediction.

We propose a new methodology in Figure 1, which is divided in two stages: data acquisition and computational model, see Figure 2 and Figure 3, respectively. Moreover, data acquisition is divided in three modules: data retrieval, data preprocessing and feature extraction. The computational model is composed of two modules: merge prediction and ARI forecast. Each module in each stage, data acquisition, and computation, involves additional tasks.

The next subsections describe the data sources and how the proposed methodology is applied on the data sources in Section 2.1, data acquisition is in Section 2.2, and the computational model in Section 2.3.

### 2.1. Data Sources

This section describes the two data sources used by the proposed methodology.

#### 2.1.1. ARI Dataset

The time series for ARI data for Mexico was generated using weekly epidemiological data reported by the Mexican Health Ministry (Secretaría de Salud), through the General Directorate of Epidemiology. This dataset is public and includes the weekly number of all medical encounters reported to the Health Ministry by healthcare facilities throughout the entire country for which the diagnosis corresponded to any of the following ICD-10 codes: J00-06, J20, J21, except J02 and J03 published in a weekly epidemiological report [48]. Weekly data for winter seasons included entries reported between epidemiological week 27 of 2002 and epidemiological week 26 of 2019 (publicly available at www.genomica.uaslp.mx/Research/Bioinformatics/Bioinformatics.html).

#### 2.1.2. Internet Search Term Dataset

The methodology proposes to use the behavior of online search engines by monitoring specific keywords, known as search terms employed by the general public when they search for disease information. The Google Trends tool is used to retrieve the required data from the Google search engine. All data used for research is available publicly in their respective sources.

### 2.2. Methodology: Data Acquisition

Data acquisition is the data science part of the methodology. The constituent elements and tasks of each stage are described with detail below.

The data acquisition stage involves three modules: data retrieval, data preprocessing and feature extraction. A detailed diagram of this stage is shown in Figure 2.

#### 2.2.1. Data Retrieval

ARI raw dataset. The data is extracted manually from the weekly epidemiological data reported by the Mexican Health Ministry (Secretaría de Salud), through the General Directorate of Epidemiology. The weekly total of ARI cases is recorded from 2002 to 2019.

Internet search terms. The search terms included a set of words that could be commonly used in Internet search engines by Mexicans and that would correlate with ARI cases. Only the search terms that are most related with the ARI behavior are needed; to find these terms an initial list of possible relevant terms is created by medical experts.

Find correlated search terms. These suggested search terms also included words more likely to be used by physicians, experts in epidemiology, virology, and respiratory infections. An automated correlation study is made as a first step to discard non-significant terms from the initial list of 168 suggested terms.

Obtain historical terms behavior. Using the list of suggested terms described previously, the historical search usage of the terms was requested to Google Trends (www.google.com/trends) to obtain weekly information on search term usage for Mexico using an identical time window as our Ministry of Health derived ARI data. This was queried using the NodeJS Software Development Kit (SDK) “google-trends-api” script created by Patrick Trasborg in 2016 under an MIT License (www.npmjs.com/package/google-trends-api), which deals with the request and retrieval functions. For each NodeJS request the Google Trends service returns a JSON file of search term usage. When no data was available, the retrieved JSON file was found to be empty.

#### 2.2.2. Data Preprocessing

Data cleaning and formatting. In the data preprocessing stage the JSON files are parsed and used to populate tables. The tables are then cleaned of missing values by filling empty cells with zeros. For the historical usage of search terms and ARI raw data, the 53rd week was eliminated for those years that had an additional epidemiological week, this to ensure a constant size of 52 weeks for all years.

Pearson correlation analysis. An automated correlation study is made as a first step to discard non-significant terms from the initial list of 168 suggested terms. Removing the terms least correlated with ARI data by using the Pearson Correlation. The weekly usage for each search term was paired with the ARI data to measure their correlation. After this process, the Google Trends data were stored along with the ARI dataset into one single data table.

Search term removal. During the selection process, terms with a correlation coefficient below 0.75 were removed, thereby reducing the list of terms. No negative correlations were included, as it was not expected for people to stop searching a specific term during an outbreak.

#### 2.2.3. Feature Extraction

In this module, the ARI dataset was analyzed to extract relevant information such as the endemic channel calculation and our proposed sum-of-sines endemic channel calculation. On the other hand, the search terms were tested with a greedy algorithm to select only the most relevant search terms.

Endemic Channel. Surveillance of infectious diseases commonly relies on the use of endemic channels to predict outbreaks based on the identification of cases which fall outside of the expected behavior, which in turn is derived from historical time series [49,50]. Endemic channels are obtained by calculating the 5th, 50th, and 85th percentiles of a disease for each week from the 5 previous years (ignoring years affected by outbreaks); the 5th percentile represents the minimum number of cases expected (area of success), the 50th percentile is the expected behavior (the median or secure area), and the 85th percentile is the risk area (area of alert, or the values pointing to possible outbreak). As such, endemic channels represent the boundaries for the expected number of cases at a given time. The occurrence of cases above this moving threshold is considered an outbreak of a disease [51].

**Figure 4:**
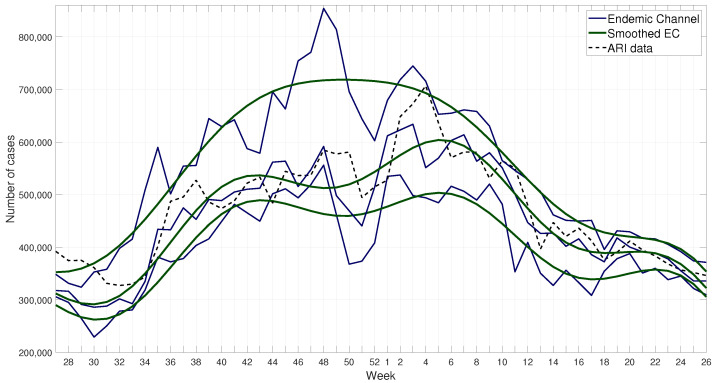
Smoothed endemic channels using sum of sines (SoS). At the top are the 100th percentiles, at the middle are the 50th percentiles and at the bottom the 1st percentiles. The thick lines show the smoothed channels (SoS), and the dotted line the acute respiratory infection (ARI) data.

Endemic Channel Smoothing. As the commonly used endemic channels have many sudden variations in their behavior, and after exploring different possible solutions [49,50], it was decided to propose a smoothed endemic channel by using the sum of sines function as a signal smoothing method. This method was applied to the 1st, 50th, and 100th percentiles of the ARI time series, resulting in a set of smoothed endemic channels, see Figure 4.

Sum of sines (SoS). A sum of sines function can be used to model time series with historical data and project its possible future behavior. Depending on such behavior, the function may be composed by an addition of several sinusoidal functions in order to fit it. A generalization is shown in the following equation:

(1) $$y=a_{1}sin\left(b_{1}x+c_{1}\right)+a_{2}sin\left(b_{2}x+c_{2}\right)+\ldots +a_{n}sin\left(b_{n}x+c_{n}\right)$$

where $a_{i}$, $b_{i}$, $c_{i}$ are unknown parameters and need to be fitted to the data; *x* represents the week number (time); *y* represents the output, i.e., the smoothed endemic channel. A non linear least squares method can be used to find values for these parameters, the fit function in MATLAB^®^ from Curve Fitting Toolbox^™^ is used for this purpose (see Figure 4), the scripts are available under request.

*Greedy Search Term Removing*. As the removal of irrelevant search terms helps minimize the input noise fed to the forecasting models, greedy search term removal was implemented [52,53]. Although brute force algorithms test all possible combinations of search terms, they are computationally intensive. The greedy algorithm selects the optimal search terms based on the root mean squared error (RMSE) between the ARI cleaned data (hereafter ARI data, cleaned during the preprocessing module) and the output of an artificial neural network.

**Figure 5:**
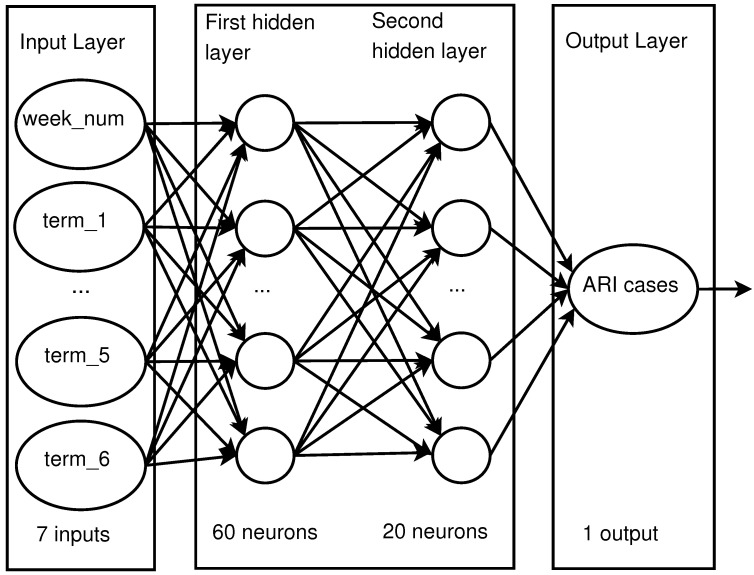
Feedforward Neural Network (FFNN) architecture.

*Feed Forward Neural Network (FFNN)*. FFNNs are also known as backpropagation networks [54,55,56]. They are composed by an input layer, one or more hidden layers, and an output layer. Each layer is composed by a set of nodes, artificial neurons. Every layer is connected to the next by weights and each neuron of a layer is connected to all the neurons on the next layer. The neurons in the hidden layers have an activation function which is applied to the inputs received from the previous layer each one multiplied by its respective weight. The number of hidden layers and neurons on each of them was estimated by a grid search algorithm that tested all configurations for one and two layers with 10 to 100 neurons on each. The best configuration found, lowest RMSE in the testing stage, is a two hidden layer architecture with 60 and 20 neurons respectively, see Figure 5. By using the fitnet function in MATLAB^®^ from the Deep Learning Toolbox^™^, a neural network was trained and tested 300 times using as input the week number and each of the terms and the average RMSE for every term was recorded; the scripts will be available under request. Subsequently the RMSE was compared between search terms, and the term with the lowest RMSE is kept. The test is repeated adding another term, until minimal or no improvement is found. Using this approach, the list of search terms was reduced to six keywords: asthma, bronchitis, what is flu, respiratory, cough, and respiratory tract (in Spanish correspond to ‘asma’, ‘bronquitis’, ‘que es la gripe’, ‘respiratorias’, ‘tos’, and ‘vias respiratorias’, respectively). These terms showed the most significant relation with ARI data.

### 2.3. Methodology: Computational Model

The second stage, the computational model, has two modules: merge prediction and ARI forecast, see Figure 1 and Figure 3. Merge prediction is divided in three tasks: (a) a forecasting model build with FFNN; (b) a projection model based on SoS; and (c) the merge prediction, which integrates both previous components and the smoothed endemic channels as inputs, see Figure 3. The forecasting model makes its predictions based on the search terms behavior, while the projection model works in parallel with ARI data and the smoothed endemic channel, and both making a one-week projection of its expected behavior.

#### 2.3.1. Forecasting Model: FFNN

**Figure 6:**
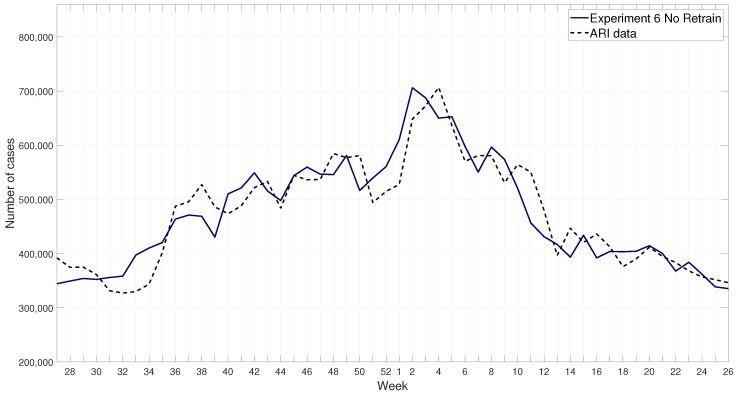
ARI data and FFNN response forecasting one week in advance for the 2017–2018 winter season.

The FFNN receives as input the current week number (1-52) and internet usage for each of the six search terms during that week, and it is trained to predict the ARI data for the next week. An example of the response of the FFNN for the 2017–2018 winter season is shown in Figure 6.

#### 2.3.2. Projection Model: SoS

**Figure 7:**
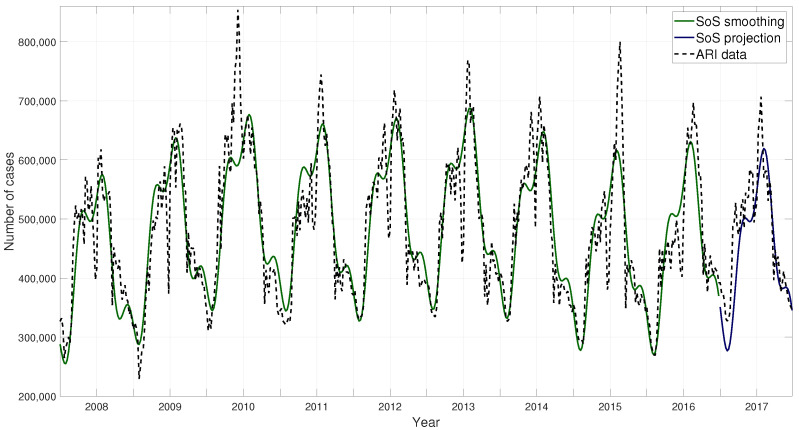
ARI data and sum of sines (SoS) fitted model.

**Figure 8:**
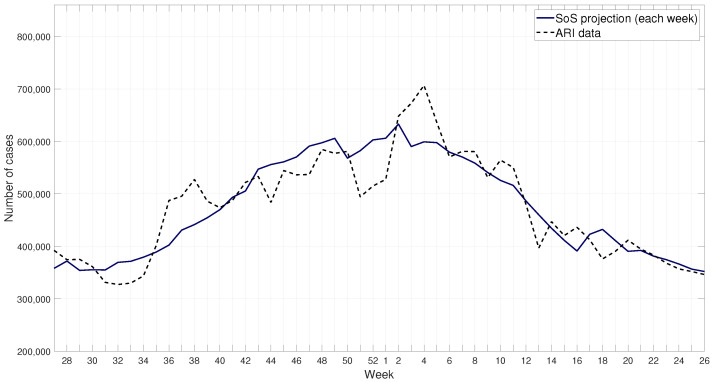
ARI data and sum of sines one-week projection for 2017–2018 winter season.

A sum of sines function was used to model the behavior of the ARI cases throughout the years. Most specifically, a five term sum of sines function was used, as denoted in Equation (1). The resulting parameters obtained by the fit function are shown in Table 1. The fitted model is then used to project its behavior. Several tests were performed in order to define the time span that results in the best prediction, leading to our adoption of a one week forward setup for our projections. The resulting fitted model for the ARI data is shown in Figure 7 and the one-week projection for the winter season 2017–2018 in Figure 8.

#### 2.3.3. Merge Prediction

For the merge prediction, a linear equation was used. This equation involves the results from the forecasting model and the projection model along with the smoothed endemic channel, as a weighted sum.

(2) $${\hat{y}}_{t}=w_{1}x_{1_{t}}+w_{2}x_{2_{t}}+w_{3}x_{3_{t}}+w_{4}x_{4_{t}}$$

where, $x_{n_{t}}$ is each of the responses, forecasting model, projection model, lower endemic channel value, and upper endemic channel value, respectively. Parameters $w_{1}$ to $w_{4}$ are the weights given to each response and they are calculated fitting the equation to minimize its RMSE using the responses and the ARI data from the immediate previous year; the resulting values are shown in Table 2.

#### 2.3.4. ARI Forecast

This module, which is part of the computational model of the proposed methodology (see Figure 1 and Figure 3), integrates all forecasting outputs from the proposed methodology, i.e., FFNN, SoS, and merge. At results section, in Figure 12 are shown the results from the ARI forecast: FFNN (Exp6), SoS, and merge.

### 2.4. Metrics

Metrics that have been reported previously in similar applications were used in order to measure the results of the Computational model [31,32], including the Pearson correlation coefficient (*r*), root mean square error (RMSE), root mean squared percent error (RMSPE), and the maximum absolute percent error (MAPE). Each of which are described below.

#### 2.4.1. Pearson Correlation

Additionally, known as bi-variate correlation, it is widely used to measure the linear correlation between two variables. The result obtained in the Computational model for each week was paired with the ARI data to measure their correlation as a metric to measure the accuracy of the forecasting model.

#### 2.4.2. Root Mean Square Error (RMSE)

RMSE is a measure of the distribution of residuals and reflects how data concentrates around the predicted model. This metric is also used to choose the best fitting network in our proposed model.

#### 2.4.3. Root Mean Squared Percent Error (RMSPE)

RMSPE measures the difference between predicted and real values as a percentage.

(3) $$\mathrm{RMSPE}=\sqrt{\frac{1}{n}\sum {\left(\frac{y_{i}-x_{i}}{y_{i}}\right)}^{2}}\times 100$$

#### 2.4.4. Maximum Absolute Percent Error (MAPE)

MAPE is most commonly used to evaluate forecasts. It has valuable statistical properties. It makes use of all observations and has the smallest variability from sample to sample. MAPE is also often useful for purposes of reporting, because it is expressed in generic percentage terms. It has some set backs with outliers, but it is still a common metric for forecasting models [57].

(4) $$\mathrm{MAPE}=\left(\underset{i}{max}\frac{\left|y_{i}-x_{i}\right|}{y_{i}}\right)\times 100$$

Unusually large errors can affect MAPE and RMSPE. However, they share some useful characteristics, since the denominator is the real expected value, the result is not affected by the unit of measurement of the series. This makes the MAPE and RMSPE a good metric for comparing the performance of a forecasting method on different series or the performance of many methods on one series. The RMSE was used to compare the method within the methodology but, as this metric is bound to the unit of measurements, it could not be used to compare with other reported models.

## 3. Results

This section presents the results from the proposed methodology fed with ARI dataset and Internet search terms. The recorded results are focused on the last stage of the methodology, the computational model, i.e., forecasting model (FFNN), projection model (SoS), and the merge prediction.

### 3.1. Forecasting Model

The FFNN was tested to assess one-week-in-advance forecasting of the ARI data for the winter seasons encompassed between 2015 and 2019 in a yearly fashion. Data from 2008 to 2018 were used in order to train the models, i.e., data from winter seasons between 2008 and 2015 were used as training data to forecast the 2015–2016 winter season.

In order to evaluate the accuracy of the forecasting model, several experiments (Exp 1 to 6) were performed. These included assessment of the training window size, as well as evaluation of the FFNN with and without retraining of the network every 13 weeks (a quarter of a year, denoted as Q1, Q2, Q3, and Q4 in Figures).

Table 3 and Table 4 show the details of the training and testing sets for all experiments to determine the best window length. Since the initial weights of the FFNN (parameters) are initialized randomly, each experiment was executed one thousand times creating the same number of networks, then each FFNN was tested with data from the 27th week of the starting year to the 26th week of ending year. The training concluded when the best FFNN was selected (the one with the lowest RMSE) to be used in the final testing. It is worth noting that the year 2009 was omitted from these tests because of the Influenza AH1N1 pandemic.

The final testing was divided in quarters, each of them composed by 13 weeks, but the results are grouped by year. On the retrained version of the tests, the network was retrained by adding the most recent data along with part of the previous training data. The version without retraining used the same network during the four quarters of each winter season test.

**Figure 9:**
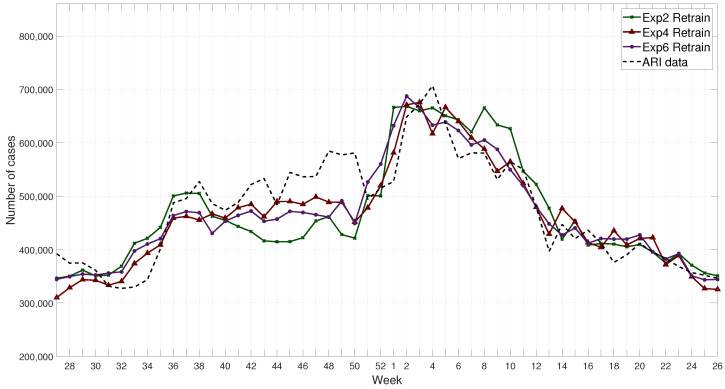
Example of results for retrained networks for the 2017–2018 winter season compared with ARI data.

**Figure 10:**
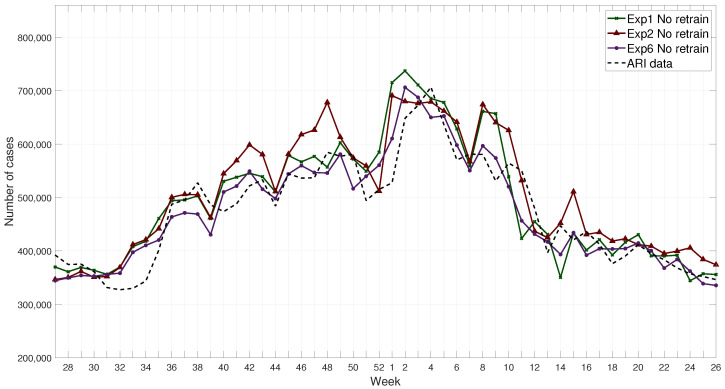
Example of results for networks without retraining for the 2017–2018 winter season compared with ARI data.

The metrics of the results are shown in Table 5, where the best three results for each of them are in bold fonts. An example of the results for the retrained FFNN is shown in Figure 9, and without retraining in Figure 10. These results show that Exp 6 without retraining have consistently more accurate results compared to other experiments.

### 3.2. Merge Prediction

**Figure 11:**
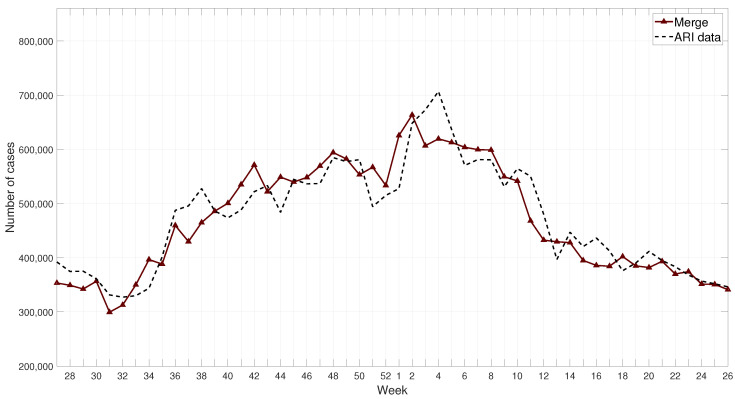
Example of results for the merge prediction for the 2017–2018 winter season compared with ARI data.

**Figure 12:**
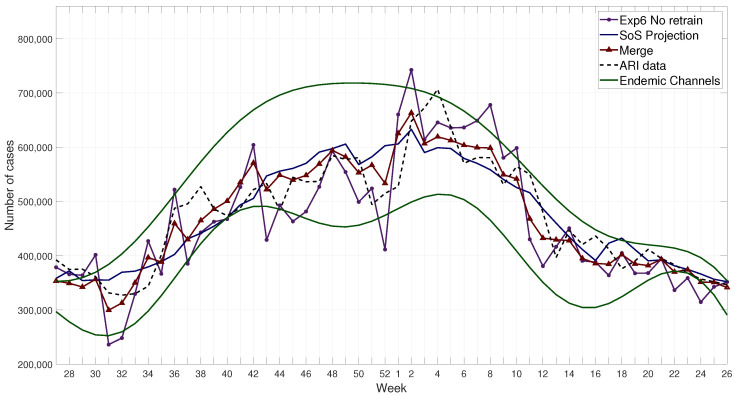
Example of all results from the model for the year 2017–2018 compared with the real values for ARI cases.

The merge prediction is composed by the forecasting model (FFNN), the projection model (SoS), and the endemic channels merged in a linear equation that reduces the error-based metrics. Results of this prediction for the 2017–2018 season are shown in Figure 11; these results can be compared with those obtained by the FFNN model, the SoS model, and the endemic channels in Figure 12. The model was tested for several seasons, 2015 to 2019, see detailed results at Appendix A.

This merged result has a reduced RMSE, RMSPE, and MAPE and a higher correlation coefficient compared with FFNN and SoS individually. The average results for these metrics obtained with the three methods during the four study seasons are shown on Table 6. This proposal is also compared with similar models found in the state of the art, using data from Google searches [32] and Twitter [31,32] for Influenza surveillance and ILI forecasting with machine learning algorithms, such as, AdaBoost, support vector machines (SVM), with linear and radial basis functions (RBF), and long short-term memory networks (LSTM), the best one-week forecast results were selected to compare with this methodology, see Table 6. It is worth noting that the RMSE is not a normalized metric, because it depends on the units of measurement, and therefore it cannot be used as comparison metric.

## 4. Discussion

Over the last century the availability of vaccines and antibiotics has resulted in a significant reduction of the impact of infectious diseases in the human population worldwide. Nevertheless, infectious diseases continue to cause significant morbidity and mortality. Of special importance, the occurrence of epidemics and pandemics has resulted in major loss of life, health system saturation, and economic burdens. Early identification of outbreaks and epidemics is considered essential in order to limit the spread and effects of an infection within a community or country. Epidemiological surveillance systems are essential tools to identify the onset of outbreaks. Surveillance systems can rely on information that is obtained actively or passively. Active surveillance systems require that epidemiological information be obtained based on case finding activities which may require identifying individuals that fulfill certain case definitions or carrying out laboratory tests to detect a specific pathogen. In contrast, passive surveillance may use routinely gathered information for analysis. As a result, active surveillance systems tend to be more complete but more expensive than passive systems [58]. In both instances, analysis of the information that has been gathered is a key element in order to identify changes in disease occurrence that indicate the onset of an epidemic in comparison to fluctuations that may be considered as normal. Of interest, current computing power allows the analysis of large data sets and with the use of diverse algorithms it is possible to identify signal changes that under traditional epidemiological analyses might be difficult to observe. In addition, the expanding use of the internet has resulted in the potential use of temporal and geographic query patterns for infectious disease surveillance [59]. As a result, over the last decade there has been an increasing interest in the development of internet usage patterns to analyze infectious diseases dynamics and forecast expected behaviors and the occurrence of epidemics [60].

In the present work we describe a computational model for ARI surveillance that might allow for early detection of outbreaks. Our model is based on ARI data reported on a weekly basis to the Health Ministry in Mexico as well as the number of internet searches of a set of terms by Mexican Google users. The model has been tested with historical data, and proved to predict the behavior of ARI data for four successive winter seasons (2015–2019); the best MAPE results being obtained with the SoS Projection and the merge prediction with 21.7% and 30.9%, respectively. In order to assess these results, we contrasted several metrics (Pearson correlation, RMSPE, and MAPE) with those reported with the use of other methodologies that have analyzed the behavior of respiratory infectious diseases. Unfortunately, there are no previous studies that have focused on ARI which provide similar metrics to assess the accuracy of forecasting, nor encompassing the same geographical and temporal boundaries of our study. Therefore, we included studies that have assessed specific respiratory infections (such as influenza or influenza-like illness) which have shown very good results [31,32]. The performance of this methodology was competitive in comparison to results reported in those studies with the use of other forecasting methodologies. The modular structure of the proposed model enables to change the forecasting or projection model to enhance the results; in addition, a decision-making stage could be added in which the predictions are analyzed to detect and send alerts when a potential outbreak is identified.

The main advantage of the proposed model is the use of data that is readily available, such as Internet search terms and routine disease surveillance data (ARI data) to predict an infectious disease. Some of previous reports that describe forecasting of respiratory infections (such as influenza infections) rely on samples obtained for virological testing rather than syndromic clinical reports [61]. While our model could be limited when assessing the behavior of a specific microorganisms (such as influenza), it could allow for the timely identification of outbreaks when the etiological agent is unknown, such as the appearance of unusual cases of pneumonia late in 2019 in Wuhan, China, which were subsequently identified as caused by a novel viral strain (SARS-CoV-2) [11]. In addition, because our system is based on routinely obtained information and does not require specific laboratory tests, it is expected to allow for surveillance of wide geographical areas, even in regions where laboratory facilities are not available. This could have immediate application in epidemiological surveillance, as a complementary methodology to already established strategies (for example, in the current SARS-CoV-2 pandemic) [62,63]. This methodology could be adapted for use at a subnational level (such as at regional or state level). Overall, the expected usefulness of ARI analysis using this methodology includes the timely identification of an increase in the number of ill persons. Of note, we observed that FFNN response forecasting (Exp6 no retrain) performed well in relation to peak number of ARI. A limitation to this observation is that our study included forecasting only for four winter seasons to be certain that this finding is reproducible in all seasons. Appropriate interventions when outbreak signals are identified by this methodology could include targeted laboratory testing, institution of outbreak assessment and control measures, as well as mobilization of health-care supplies (such as medications or vaccines, when available). In addition, it can be adapted to be used in other countries. This may be of particular use in regions where surveillance systems require strengthening. Furthermore, this proposal could also be used for assessment of other infectious diseases that show seasonal patterns, such as gastrointestinal infections, dengue, and varicella. The inclusion of an automatic strategy for term removing is also an advantage, since this allows to update the search term list and allows for inclusion of many additional terms, as the number of terms in the initial list does not matter because the model will reduce it to the minimum required for predicting results, eliminating subjectivity; nevertheless, the time required to collect and analyze a long list of new search terms would require additional time. During the first stage of the development of our model, Google Correlate was used to obtain the initial list of potential search terms analyzing their correlation with ARI data; unfortunately, this tool was discontinued at the end of 2019. Nevertheless, potential search terms can be assessed with the use of correlation tests, such as Pearson’s correlation. For the feature extraction stage, we assessed several approaches for endemic channel smoothing, including polynomial, splines, Fourier, among others [49]. In addition, several techniques to model the cyclic behavior of time series on ARI data were explored, and found that SoS is simple, and resulted in improved data fitting. Moreover, other techniques were explored to model the cyclic behavior, such as the Holt–Winters method which is used in economy; however, they are more complex and did not improve the model.

## 5. Conclusions

This work introduces a new methodology for infectious disease forecasting using ARI data and Internet search terms. The results show that the combination of different data analysis techniques (FFNN, SoS, and smoothed endemic channels) can provide an accurate prediction for ARI data one week in advance. The final model could be used along with the endemic channels to detect possible outbreaks. The methodology can be expanded to work by regions (or states), to analyze data from other countries, or to assess the behavior of other seasonal infectious diseases. In addition, this proposal could allow the Mexican health authorities to complement traditional surveillance methods for the timely identification of ARI outbreaks. This would be a relevant application of this model, since early detection and response to an outbreak can decrease the costs and the impact associated with it.

## Figures and Tables

**Table 1:** Values of the adjusted parameters using the non linear least squares fitting method. Each row *i* represents the parameters affecting the *i*th sine.

*i*	*a*	*b*	*c*
1	21,680.0	0.002802	0.316
2	8886.0	0.004113	2.828
3	4021.0	0.120700	1.491
4	746.3	0.023590	1.264
5	1155.0	0.362400	−0.689

**Table 2:** Parameter values using responses from the 27th week of 2017 to 26th week of 2018.

Parameter	Value
$w_{1}$	0.370
$w_{2}$	0.045
$w_{3}$	0.380
$w_{4}$	0.205

**Table 3:** Training sets. Each test consisted of twelve experiments, six with retraining and six without retraining the network.

FFNN	Start Year (On Week One)	End Year (On Week 26)	Window (Years)
Exp 1	{2008,2010,2011,2012}	{2015,2016,2017,2018}	6.5
Exp 2	{2010,2011,2012,2013}	{2015,2016,2017,2018}	5.5
Exp 3	{2011,2012,2013,2014}	{2015,2016,2017,2018}	4.5
Exp 4	{2012,2013,2014,2015}	{2015,2016,2017,2018}	3.5
Exp 5	{2013,2014,2015,2016}	{2015,2016,2017,2018}	2.5
Exp 6	{2014,2015,2016,2017}	{2015,2016,2017,2018}	1.5

**Table 4:** Testing sets. Each test consisted of twelve experiments, six with retraining and six without retraining the network.

FFNN	Start Year (On Week One)	End Year (On Week One)
Exp 1	{2015,2016,2017,2018}	{2016,2017,2018,2019}
Exp 2	{2015,2016,2017,2018}	{2016,2017,2018,2019}
Exp 3	{2015,2016,2017,2018}	{2016,2017,2018,2019}
Exp 4	{2015,2016,2017,2018}	{2016,2017,2018,2019}
Exp 5	{2015,2016,2017,2018}	{2016,2017,2018,2019}
Exp 6	{2015,2016,2017,2018}	{2016,2017,2018,2019}

**Table 5:** Averaged metrics for all experiments with and without retraining. Best values are in bold fonts.

FFNN	RMSE	CORR	RMSPE	MAPE
Exp 1 Retrain	86,139.27	0.72	19.76%	54.09%
Exp 2 Retrain	89,181.04	0.72	19.35%	49.98%
Exp 3 Retrain	86,744.66	0.70	19.46%	54.73%
Exp 4 Retrain	80,932.07	0.77	18.60%	51.83%
Exp 5 Retrain	80,915.79	0.77	18.56%	49.91%
Exp 6 Retrain	**74,482.54**	0.79	**17.42%**	**45.50%**
Exp 1 No retrain	86,875.26	**0.94**	20.07%	52.46%
Exp 2 No retrain	87,912.19	**0.93**	19.68%	50.91%
Exp 3 No retrain	90,353.82	0.90	20.55%	55.80%
Exp 4 No retrain	82,922.59	**0.92**	19.20%	47.68%
Exp 5 No retrain	**80,360.40**	0.76	**18.49%**	**47.43%**
Exp 6 No retrain	**74,169.01**	0.82	**17.35%**	**42.38%**

**Table 6:** Comparing the merge prediction with other methods. Metrics are calculated averaging consecutive testing seasons.

Model	Reference	CORR	RMSPE(%)	MAPE(%)	Averaged Seasons
Exp 6	This model	0.80	16.48%	50.10%	2015–2019
SoS Projection	This model	0.88	**10.92%**	**21.70%**	2015–2019
Merge	This model	0.88	**11.27%**	**30.90%**	2015–2019
SVM (RBF)	[32]	**0.89**	26.90%	137.00%	2013–2015
AdaBoost	[32]	**0.92**	16.10%	40.90%	2013–2015
SVM	[31]	**0.90**	**10.43%**	**31.84%**	2011–2014
AdaBoost	[31]	0.87	13.35%	43.61%	2011–2014
LSTM	[31]	0.61	15.46%	46.67%	2011–2014

**Figure A1:**
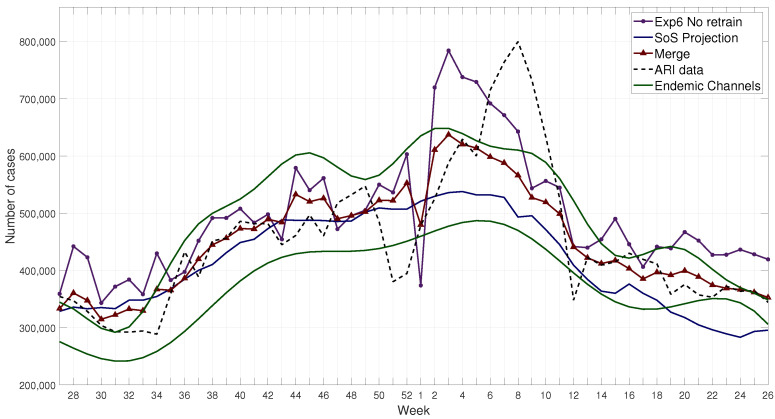
Results from the model for the year 2015–2016 compared with the real values for ARI cases.

**Figure A2:**
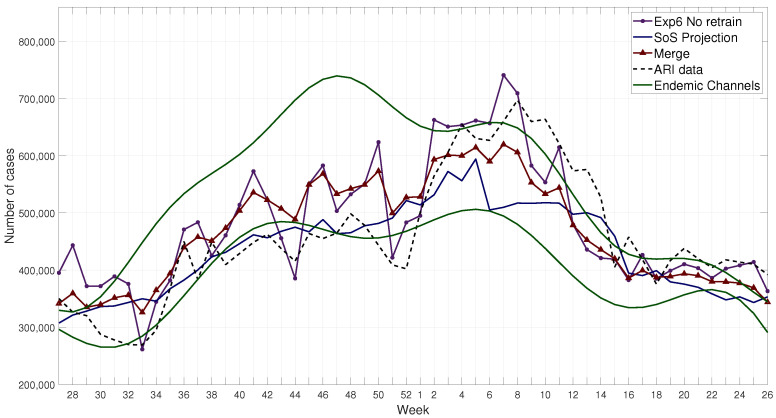
Results from the model for the year 2016–2017 compared with the real values for ARI cases.

**Figure A3:**
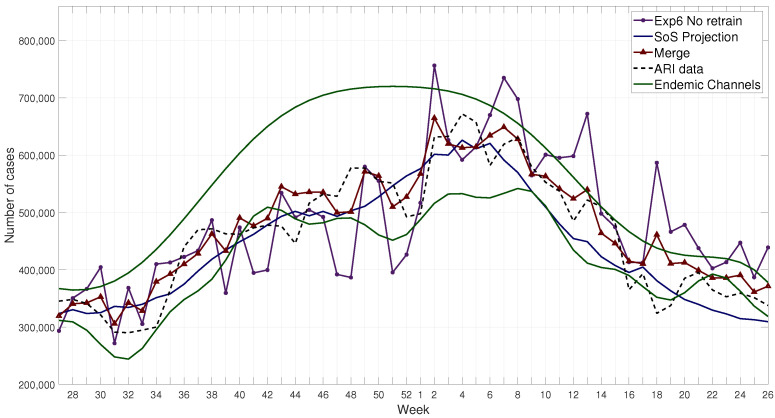
Results from the model for the year 2018–2019 compared with the real values for ARI cases.

## Appendix A. Results Obtained for 2015–2016, 2016–2017, and 2018–2019 Seasons

The model was tested for several seasons, from 2015 to 2019, see Table 6. Throughout the paper, we only show plots with results from the 2017–2018 season, see Figure 6, Figure 7, Figure 8, Figure 9, Figure 10, Figure 11 and Figure 12. Here we show the results from the seasons 2015–2016 (Figure A1), 2016–2017 (Figure A2), and 2018–2019 (Figure A3). Additionally, Table A1 shows the metrics for each season.

**Table A1:** Detailed metrics for each season from 2015 to 2016.

	RMSE	CORR	RMSPE	MAPE
**2015–2016**				
Exp 6	90,666.4436	0.78	20.1%	52.0%
SoS Projection	87,711.5075	0.85	12.5%	31.0%
Merge	70,084.2756	0.84	13.5%	35.0%
**2016–2017**				
Exp 6	69,404.4634	0.82	16.1%	39.0%
SoS Projection	67,787.1978	0.84	12.1%	25.0%
Merge	67,205.4062	0.82	12.9%	30.0%
**2017–2018**				
Exp 6	60,084.4026	0.86	11.2%	28.0%
SoS Projection	42,529.7762	0.91	8.8%	15.0%
Merge	38,142.7486	0.93	7.8%	17.0%
**2018–2019**				
Exp 6	83,400.9898	0.75	18.4%	81.0%
SoS Projection	45,056.3245	0.92	10.1%	16.0%
Merge	42,022.3458	0.94	10.7%	42.0%
**Averaged**				
Exp 6	75,889.0749	0.80	16.4%	50.0%
SoS Projection	60,771.2015	0.88	10.9%	21.7%
Merge	54,363.694	0.88	11.2%	30.9%
